# Debilitating Musculoskeletal Disease in Two Free-Ranging Juvenile American Black Bears (*Ursus americanus*)

**DOI:** 10.3390/ani14142088

**Published:** 2024-07-17

**Authors:** Isabella C. Fahrenholz, Michelle M. Dennis, Federica Morandi, Keren E. Dittmer, Julie D. Sheldon

**Affiliations:** 1 Department of Small Animal Clinical Sciences, University of Tennessee College of Veterinary Medicine, Knoxville, TN 37996, USA; ifahrenh@vols.utk.edu (I.C.F.); fmorandi@utk.edu (F.M.); 2 Department of Biomedical and Diagnostic Sciences, University of Tennessee College of Veterinary Medicine, Knoxville, TN 37996, USA; mdenni12@utk.edu; 3 School of Veterinary Science, Massey University, Palmerston North, NZ 4410, USA; k.e.dittmer@massey.ac.nz

**Keywords:** American black bear, chondrodysplasia, epiphyseal dysplasia, joint laxity, osteoporosis, *Ursus americanus*, vitamin D

## Abstract

**Simple Summary:**

This case report highlights two wild juvenile American black bears that were admitted to a rehabilitation facility but were euthanized due to severe immobility and joint laxity. Known musculoskeletal diseases of young bears are usually caused by trauma in the wild, or potentially rickets (a form of malnutrition) if raised in captivity. However, Case 1 was diagnosed with abnormal cartilage development (chondrodysplasia), likely of genetic origin, and Case 2 had osteoporosis and unidentified collagen abnormalities which are both unique to the current literature. With growing black bear populations, and increasing wildlife–human interface, we advise wildlife biologists and veterinarians to monitor and evaluate similar cases to help us further understand these uncommon diseases.

**Abstract:**

Severe musculoskeletal disease characterized by marked joint laxity was the cause of euthanasia in two wild juvenile American black bears (*Ursus americanus*) admitted to a rehabilitation facility in eastern Tennessee in 2023. Previously, almost all reported musculoskeletal diseases in this population were of traumatic etiology, even in malnourished yearlings. Case 1 was an orphaned 11-month-old male cub exhibiting disproportionate dwarfism, progressive immobility, and joint laxity. Necropsy findings suggested either chondrodysplasia or rickets, and imaging findings supported a skeletal dysplasia. Case 2 was a 14-month-old emaciated male yearling exhibiting joint laxity and immobility. Necropsy findings showed osteoporosis and serous atrophy of fat, and imaging findings were inconsistent with a skeletal dysplasia. Both cases were clinically inconsistent with rickets based on normal calcium, phosphorous, and parathyroid hormone concentrations; however, Case 1 had hypovitaminosis D (9 nmol/L) compared to healthy juvenile black bears. We hypothesize that Case 1 had a genetic chondrodysplasia while the osteoporosis of Case 2 was due to chronic malnutrition. The goal of this case report is to inform wildlife agencies and facilities to monitor for similar, non-trauma-related debilitating musculoskeletal disease in free-ranging bears and evaluate cases that allow us to further understand the disease processes involved.

## 1. Introduction

Free-ranging juvenile (<2 years old) American black bears (*Ursus americanus*) undergoing rehabilitation in east Tennessee commonly present with musculoskeletal disease due to trauma [1]. Of 195 juvenile black bears released from Appalachian Bear Rescue (ABR) between 1996 and 2021, 9% were successfully treated for orthopedic disease, the majority being fractures [1]. Forty-nine percent of released bears were successfully treated for malnutrition, but none had secondary musculoskeletal disease. One out of 337 total bears presenting to ABR was diagnosed with shoulder dysplasia presumptively attributed to osteochondrosis [1]. To our knowledge, there have been no documented cases of developmental disease or malnutrition causing debilitating immobility and joint laxity in Ursidae. Joint laxity has been reported in cases of congenital rickets in lambs [2] and chondrodysplasia in dogs [3] and humans [4]. Malnutrition has also been speculated to cause carpal joint laxity in puppies [5] and has been associated with joint hypermobility in children [6].

American black bears (*Ursus americanus*) are opportunistic omnivores with their diet consisting mostly of vegetation, insects, and less commonly other animals [7]. Bears living near human and agricultural activities are found to also consume human-related foods [8]. Breeding season occurs in the summer with a 6-month gestation [9]. In the winter, American black bears remain dormant in dens for 3–6 months depending on latitude [10]. Pregnant females give birth to altricial cubs in January–February [11] after delayed implantation [9]. Cubs nurse in the den for the first few months of life [9] until emerging in spring. Families nurse and forage together throughout summer and fall, followed by denning in the winter. Mothers generally provide care for their cubs for 16–17 months and yearlings separate between May and June after their second winter [12]. Threats to American black bears are primarily anthropogenic (vehicular trauma and human-related conflicts), and cubs are often orphaned as a result. However, when placed in rehabilitation facilities, young bears have relatively high rates of successful release [1].

Appalachian Bear Rescue (ABR) is a juvenile black bear rehabilitation facility located in Townsend, Tennessee. Bears are rescued by state and national wildlife agencies and aged based on time of year and presence (>1 yr; yearling) or absence (<1 yr; cub) of adult canine teeth. Bear birth dates are standardized as born on January 17 of each year and bears presenting prior to April 15 are considered neonates [1]. Bears at ABR are cared for by curators and monitored via continuous video footage 24 h per day and veterinary care is provided by the University of Tennessee College of Veterinary Medicine Zoological Medicine Service immediately upon intake and throughout their stay as needed. Neonates are housed in veterinary cages in climate-controlled buildings until healthy and mature enough to move to indoor acclimation enclosures. The indoor enclosures are 10’.0″ × 10’.0″ and include sliding doors to allow access to multiple rooms. Cubs of similar size are housed together, and various enrichment is provided daily (climbing structures, branches, hidden food). Bears experience a natural light cycle via large windows. Once cubs are acclimated to the indoor enclosure, they are given access to a fenced-in outdoor enclosure of similar area and are eventually transitioned to a completely outdoor 0.2-hectare natural forest enclosure lined by 3 m tall chain link electric fencing covered with a dark tarp to serve as a visual barrier, and it includes natural ground cover and trees for climbing. Naturalistic enclosures also contain plastic pools (2.5 m diameter) for wading and other sources of enrichment (car tires, hanging ropes, wooden platforms), and can house up to 10 bears at a time. Regarding feeding, neonates are bottle-fed until able to lap formula from a bowl (~3 months old) and transitioned to solid foods. Bears are fed commercially available pellets (Mazuri Bear Diet, Purina Mills, Gray Summit, MO, USA), dried mealworms (Hatortempt, Superior Pet Supplies Inc., Lichen discount, Xiquanlu county, Shandong, China), and puppy chow (Purina Complete Chicken & Rice, St. Louis, MO, USA). Bears also receive seasonally available natural foods such as blueberries (*Vaccinium* spp.), blackberries (*Rubus* spp.), apples (*Malus* spp.), peanuts (*Arachis* spp.), and acorns (*Quercus* spp.). Diet items are thrown into the enclosure every 1–2 days to prevent human–food association and encourage foraging.

This report summarizes two cases of severe musculoskeletal disease with marked joint laxity in juvenile American black bears which have yet to be reported in the literature. This case report aims to discuss the potential etiologies of these unique cases with the goals of informing wildlife agencies and facilities to monitor for similar musculoskeletal diseases in free-ranging bears and evaluate cases that allow us to further understand the disease processes involved.

## 2. Case 1

A 5 kg, 11-month-old male American black bear was one of three orphaned cubs rescued by Appalachian Bear Rescue (ABR) after the mother was euthanized due to vehicular trauma in Gatlinburg, Tennessee in December 2022. Intake examinations were performed at the University of Tennessee Veterinary Medical Center (UTVMC). All bears in this report were anesthetized with the same protocol: induction via intramuscular xylazine (2 mg/kg, 100 mg/mL, AnaSed ^®^, VetOne, Boise, ID 83705, USA) and ketamine (5 mg/kg, 100 mg/mL, Zetamine ^®^, VetOne, Boise, ID 83705, USA), maintenance with inhalant isoflurane (Piramal Pharma Limited Digwal Village, Kohir Mandal, Sangareddy District, Telangana State, India) via facemask titrated between 1 and 5%. On initial examination, Case 1 was proportionately smaller than the two siblings (Case 1 total length 73 cm, height 28.5 cm, and head width 8 cm compared to siblings’ total length 76 and 81 cm, height 40 and 38.5 cm, and head width 11 and 10 cm, respectively) and had a poor body condition (score of 3 out of 9). Case 1 also had two small subcutaneous nodules suspected to be abscesses—one in the left axilla and one on the ventral abdomen that were lanced and cleaned. This patient was treated with subcutaneous fluids (Isotonic crystalloid 60 mL/kg, Normosol-R ^®^; Hospira Inc, Lake Forest, IL 60045, USA) and a dose of an antibiotic (Ceftiofur 7 mg/kg, 100 mg/mL, subcutaneously, crystalline free acid, Excede ^®^, Zoetis, Parsipanny, NJ 07054, USA). Complete blood count and plasma biochemistry were performed. Compared to healthy 11–12-month-old American black bear cubs [13], Case 1 had mild anemia, mildly decreased blood urea nitrogen (BUN), creatinine, total protein, albumin, and cholesterol concentrations, and decreased alanine transaminase (ALT) and alkaline phosphatase (ALP) activities (Table 1). Total calcium and phosphorus concentrations were within normal limits. The three cubs were discharged to ABR for continued rehabilitation.

**Figure 1:**
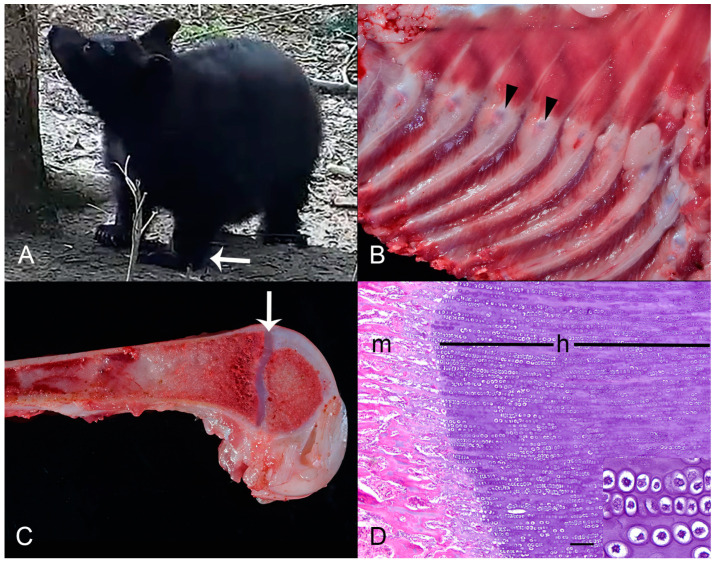
Case 1: Stature and pathological findings. (**A**) Stance as observed in enclosure. Note hyperextension of carpi (arrow) and disproportionate dwarfism. (**B**) Pleural surface of right hemithorax showing enlarged costochondral junctions (arrowheads). (**C**) Longitudinally transected distal femur with thickened physis (arrow). (**D**) Photomicrograph of distal femur physis; h = widened zone of hypertrophy (inset), m = metaphyseal bone. Hematoxylin and eosin (HE). Bar = 150 µm.

Two months after intake, the cubs were released from an indoor acclimation enclosure to an outdoor natural forest enclosure. During growth, Case 1 developed disproportionate dwarfism (i.e., shortened limbs relative to body length and width) and a wide-based stance due to excessive thoracic width relative to height. He also exhibited hyperextension of the carpi (Figure 1A), avoided climbing, showed intermittent shifting forelimb lameness, was crawling on his antebrachia, and made frequent vocalizations. The siblings remained normal.

Upon recheck examination at UTVMC in February 2023, Case 1 weighed 18 kg and despite being under a light plane of general anesthesia, physical examination (passive range of motion and palpation of bones) showed markedly painful reaction to palpation of forelimbs, especially of the shoulders. Case 1 also had severely hyperflexible appendicular joints, particularly the carpus, with excessive hyperpronation and hypersupination (Video S1) with manipulation greater than about 180 degrees in each direction without resistance. This was later compared to palpation of a healthy sibling’s carpal range of motion during its release examination, which was more rigid and less than 180 degrees of rotation (Video S2). Plasma biochemistry was repeated; compared to the December exam, notable differences included mildly elevated total calcium concentration and mildly decreased aspartate aminotransferase (AST) activity. ALP activity and phosphorus concentration were within normal limits.

**Figure 2:**
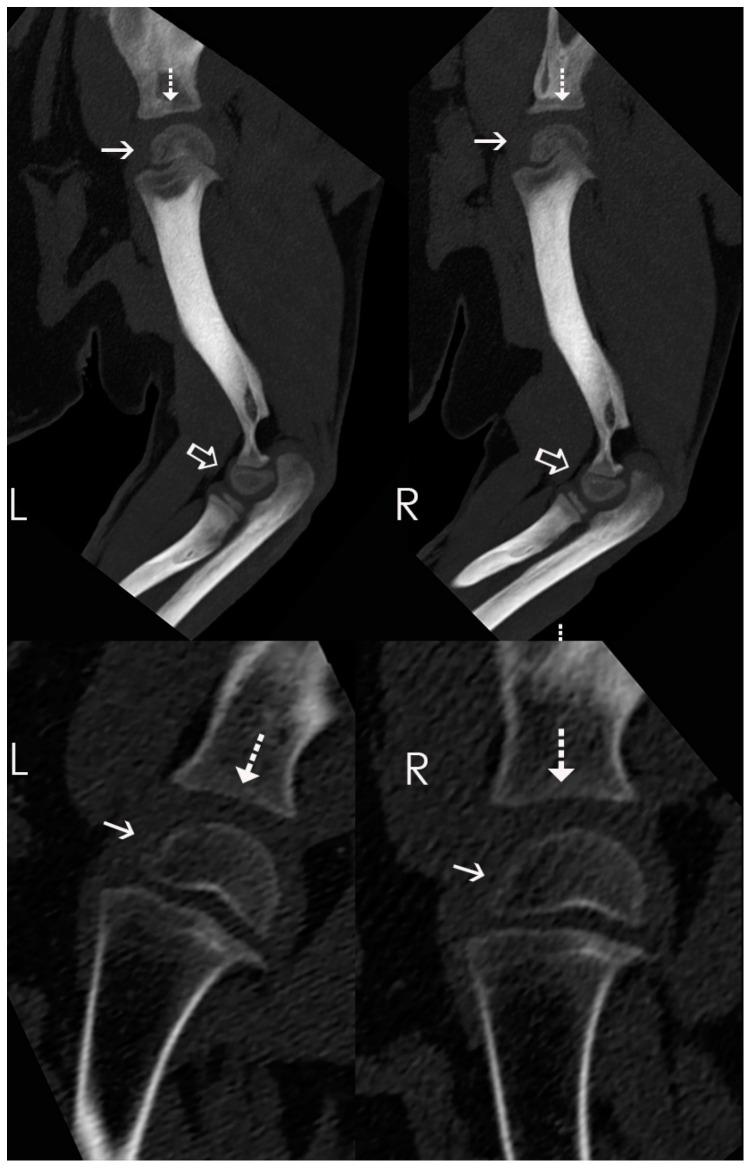
Case 1: Sagittal MIP CT reconstructions of the elbows and shoulders (top row) and parasagittal thin-section reconstruction of the shoulders (bottom row). Notice the small and incompletely mineralized proximal humeral epiphyses, with irregular cranial margins (solid arrows), and the flattened shape of the glenoid cavities (dotted arrows). The distal humeral condyles are also smaller than normal and mildly irregular (open arrows), and the proximal radial epiphyses are small and irregularly marginated. As a result of the small epiphyses, the joint spaces appear widened.

Computed tomography (CT) of the forelimbs was performed while the patient was anesthetized in sternal recumbency. The CT was performed using a Philips Brilliance 40 mCT, with acquisition at 0.9 mm collimation and reconstruction at 0.9, 1, and 2 mm collimation, and processed with bone and soft tissue algorithms. No contrast was given. Compared to other bears of similar or younger age, the glenoid cavities appeared bilaterally flattened, and the epiphyses of the humeral heads were small and had mildly irregular margination (Figure 2). The remaining epiphyses were subjectively smaller than expected, resulting in widening of the physes and joints. These combined findings were most suggestive of epiphyseal dysplasia. Based on progressive severe pain, immobility, joint laxity, and imaging findings, Case 1 was humanely euthanized (Pentobarbital, 5 mL intravenously, Fatal Plus ^®^, Vortech, Dearborn, MI, USA).

Gross necropsy showed that Case 1 weighed 18.72 kg and was in good nutritional condition. Long bones had subjectively flattened articular surfaces of epiphyses and widened joint spaces, most notably on the shoulder, suggesting mild bilateral glenoid dysplasia. Hypersupination and hyperpronation of appendicular joints were noted, most severely in the carpi, with manipulation greater than 180 degrees in each direction. The costochondral junctions were enlarged (Figure 1B), and the ribs were pliable and bent easily before breaking with manual pressure. There was subjectively marked diffuse physeal thickening (Figure 1C) of the proximal humerus, femur, and ribs with osteopenia, and subjective mild thickening of articular cartilage. A subcutaneous well-demarcated off-white nodule (2.5 × 1.5 × 0.7 cm) was noted in the mammary gland, but histopathology was not pursued due to its mild severity and at the time not apparently related to the bear’s primary conditions.

On microscopic examination, physes were thickened with a markedly widened zone of hypertrophy (Figure 1D). Metaphyseal trabecular bone had occasionally wide osteoid seams. There was mild cortical osteopenia, scattered small foci of medullary stromal proliferation, abundant osteoclastic resorption, and a potential growth arrest line in the femoral metaphysis. These combined findings were consistent with either rickets or chondrodysplasia. Secondary findings included mild proliferate esophagitis with mucosal nematode adults and ova consistent with *Capillaria* spp., moderate multifocal granulomatous mural enterocolitis, and mild focal subacute eosinophilic mural esophagitis.

## 3. Case 2

A 4.3 kg, 14-month-old male American black bear was rescued by ABR after being found emaciated and alone in the Great Smoky Mountains National Park in March 2023. Curators noted this yearling exhibited possible lameness and difficulty climbing down a tree prior to capture. Intake examination was performed at UTVMC, and the bear was determined to be markedly underconditioned, with a body condition score of 2 out of 9. Similar to Case 1, this bear presented proportionately small for its age (total length 67.5 cm, height 37 cm, and head width 13 compared to an age-matched healthy yearling with total length 124 cm, height 65 cm, and head width 20 cm). Case 2 had hyperflexible and hyperextendable appendicular joints, similar to Case 1. Case 2 also had fleas and a subcutaneous nodule in the inguinal region in which a fine needle aspirate confirmed an abscess with mixed inflammatory cells and bacteria. The abscess was lanced and flushed. Complete blood count, plasma biochemistry, total thyroid hormone concentration, and serum vitamin D profiles were performed. Compared to healthy 11–12-month-old bears [13], Case 2 had mild anemia, moderate leukopenia, mildly decreased total plasma protein concentration, marked mature neutropenia, and mild lymphopenia, eosinopenia, and monocytopenia (Table 1). In addition, there were decreases in BUN, total protein, albumin, globulins, glucose, and calcium concentrations and decreased ALP, ALT, and CK activity. Phosphorus concentration was within normal limits (Table 1). When compared to other emaciated yearling American black bears [13], Case 2 had moderate leukopenia, moderate mature neutropenia, mild panhypoproteinemia, and lower ALT, AST, CK, ALP, calcium, and phosphorus concentrations. Total thyroid hormone (T4; 2.6 μg/dL) was normal compared to adult American black bears [14].

Whole-body radiographs were performed with the patient anesthetized (same protocol previously described in Case 1). The radiographs showed open physes, incompletely ossified epiphyses and carpal and tarsal bones, and the absence of mineralized patellae; the degree of skeletal mineralization was considered to be slightly delayed for the age, possibly due to a chronic nutritional deficiency; epiphyseal dysplasia was considered much less likely. There was evidence of prior trauma, with a fracture of the right 13th rib, and a markedly narrow L3–4 intervertebral disc space. This patient was initially treated with Nitenpyram (Capstar ^®^, 11.4 mg per rectum, PetIQ Inc., Omaha, NE 68138, USA) for fleas, subcutaneous fluids (Isotonic crystalloid 60 mL/kg, Normosol-R ^®^; Hospira Inc, Lake Forest, IL 60045, USA), and calcium gluconate (50 mg/kg subcutaneously, 100 mg/mL, Fresenius Kabi, Bad Homburg, Germany) and was discharged to ABR for further monitoring.

Video footage of Case 2 showed a severely slow, cautious, and stiff gait and often reluctant to move compared to normal bears. Case 2 presented to UTVMC for a recheck examination one week after intake. Case 2 was anesthetized (same protocol previously described in Case 1), and the physical exam was similar to the initial exam. Plasma biochemistry was repeated. Compared to the intake exam, results showed mildly decreased creatinine and moderately decreased glucose concentrations, and mildly increased gamma-glutamyl transferase (GGT) activity. Total calcium and phosphorus concentrations were within normal limits. Based on progressive severe pain, immobility, and joint laxity, Case 2 was humanely euthanized.

**Figure 3:**
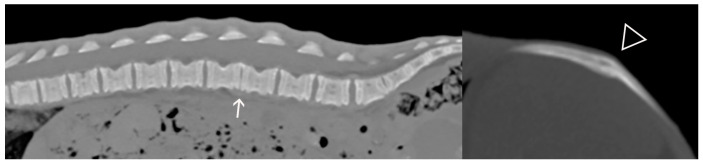
Case 2: Sagittal CT reconstruction of the spine (**left**) and oblique reconstruction of the right 13th rib, displayed in bone window (**right**). Notice the collapse of the L3–4 intervertebral disc space (solid arrow) and the smooth bridging callus at the level of a chronic rib fracture (open arrowhead). Overall bone opacity is normal.

**Figure 4:**
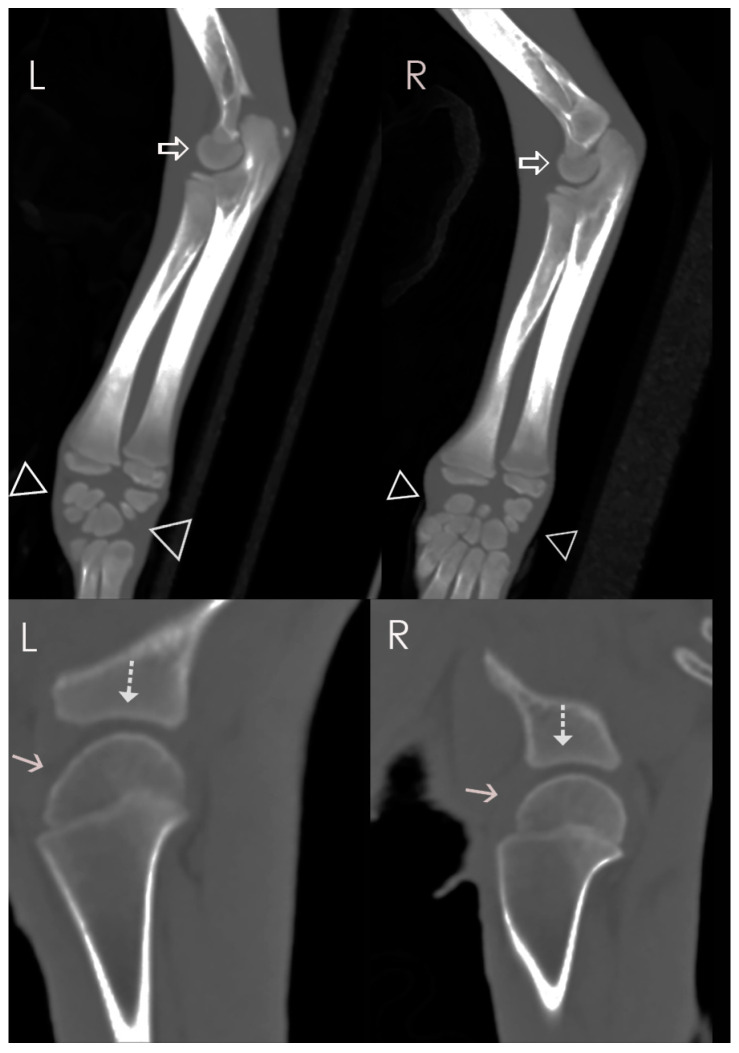
Case 2: Oblique parasagittal MIP CT reconstructions of the elbows and carpi (top row) and parasagittal thin-section reconstruction of the shoulders (bottom row). Although the proximal humeral epiphyses are incompletely mineralized, they are larger and smoother than in Case 1 (solid arrows); the glenoid cavities have a normal morphology (dotted arrows). The distal humeral condyles are normal in size and the joint space of the elbow is within normal limits. The distal physes of the radii and ulnae are only slightly smaller than expected for the age, and the carpal bones are normally mineralized (open arrowheads).

A postmortem whole-body computed tomography (CT) scan was performed using a Philips Brilliance 40 mCT, with acquisition at 0.9 mm collimation and reconstruction at 0.9 and 2 mm collimation. No contrast was given, and the data were processed with bone and soft tissue algorithms. The CT showed near-complete collapse of the L3–4 intervertebral disc space, with attenuation of the epidural fat at this site, concerning for a traumatic disc extrusion (Figure 3). A chronic, nearly healed fracture of the right 13th rib was confirmed (Figure 3). The degree of mineralization of the epiphyses and carpal and tarsal bones was considered only slightly delayed for the age, and again considered most likely to be the result of starvation (Figure 4).

**Figure 5:**
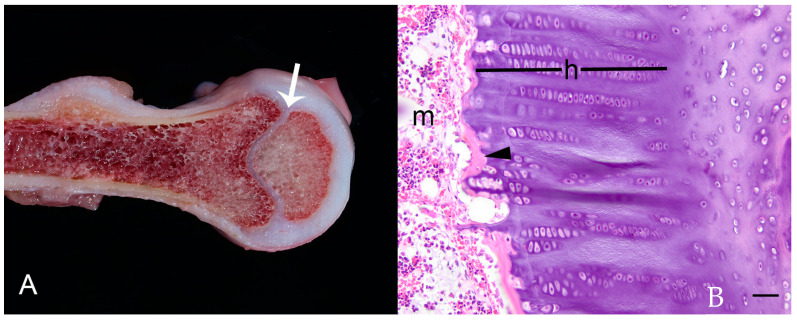
Case 2: Histopathological findings. (**A**) Longitudinally transected proximal femur with thick articular cartilage and open physis (arrow). (**B**) Photomicrograph of proximal femur physis with relatively normal thickness of zone of hypertrophy (h), metaphyseal cap of bone (arrowhead), and paucity of primary spongiosa in metaphysis (m). HE. Bar = 50 µm.

Gross necropsy showed that Case 2 weighed 4.65 kg and was in poor nutritional condition. There were several small areas of hemorrhage in the skin, subcutis, and fascia noted on the ventral midline, medial thighs, and between ribs on the right lateral thorax. Hypersupination and hyperpronation of appendicular joints were noted, particularly of the distal limbs, and most severely in the carpi. Affected joint spaces were also subjectively widened and epiphyses were comparatively small. On hemisection, all examined long bones and vertebral bodies contained open physes. The articular cartilage of long bones, most notably the femoral head, was subjectively thickened (up to 4 mm; Figure 5A). Unlike Case 1, the ribs and costochondral junctions were grossly normal, and the ribs snapped when bent, but did not seem brittle. Secondary findings included approximately 30 mL of serosanguinous fluid (presumptive transudate) in the peritoneal cavity.

Histopathology of ribs, femoral head, vertebral body, and carpal bone revealed moderate osteopenia consistent with osteoporosis. Metaphyseal physes were capped with bone and lacked primary spongiosa (Figure 5B), indicating inactive growth. Primary spongiosa were sparse and thin, with irregular cement lines (remodeling). Articular cartilage was thick, but its matrix stained evenly with Toluidine blue stain (non-degenerate). Bone marrow contained increased mature neutrophils, potentially related to a regenerative response to neutropenia and pale basophilic matrix (presumptive serous atrophy of fat). Together, these findings were not consistent with rickets or chondrodysplasia, but more likely a consequence of starvation or a possible collagen abnormality.

## 4. Retrospective Analysis of Vitamin D Profiles

Serum 25-hydroxyvitamin D (via radioimmunoassay, Immunodiagnostic Systems (IDS), United Kingdom), ionized calcium (via ion-selective electrode, NOVA analyzer, Waltham, MA, USA), and parathyroid hormone (via chemiluminescence immunoassay, IDS) concentrations were measured retrospectively from Case 1’s intake examination (banked serum stored in −80 F for 4 months) and Case 2’s recheck examination (stored frozen on ice, sent out the same day) (Animal Health Diagnostic Laboratory, College of Veterinary Medicine, Michigan State University, Lansing, MI 48909, USA; validated for dogs, cats, and horses) based on sample availability (Table 2). Additionally, these tests were performed on opportunistically collected serum during release exams of other juvenile American black bears (ranging in age from 11 to 18 months, median age 11 months) presenting to ABR during the following year. Based on these preliminary data, Cases 1 and 2 do not fit the classic rickets or secondary hyperparathyroidism pattern of increased parathyroid hormone and low vitamin D concentration. Instead, parathyroid hormone concentrations were low (Case 2) or normal (Case 1) compared to other bears, 25-hydroxyvitamin D concentration was low (Case 1) compared to other bears and domestic dogs [15], and ionized calcium values were within normal limits (Case 1 and 2) compared to other bears and domestic dogs [15]. Further investigation of normal vitamin D profiles and metabolism is needed on systemically healthy American black bears.

## 5. Discussion

ABR has been rehabilitating orphaned, injured, and ill American black bear cubs and yearlings in the southeastern United States for 27 years with a release rate of 85% and a caseload of approximately 20 bears per year [1]. Causes of death are primarily due to trauma, followed by developmental issues (i.e., hydrocephalus) and malnutrition [1]. These two cases of debilitating musculoskeletal disease were not appropriate candidates for rehabilitation and release and were thus euthanized. Clear etiologies for these musculoskeletal diseases were not apparent antemortem and were difficult to determine postmortem. Initially, with Case 1, rickets was a differential; however, most cases of rickets in the field of zoological medicine are caused by nutritional deficiency of vitamin D or phosphorus, or a lack of access to sunlight leading to vitamin D deficiency. Case 1 was with its mother up until its intake to ABR, and thus was unlikely a case of starvation and disease secondary to malnutrition. Case 2 was found emaciated and small, like the classic malnourished yearling cases seen frequently at ABR; however, the clinical signs of severe joint laxity, pain, and immobility made it unique. Initially, Case 2 was thought to have a similar disease process to Case 1, but its histopathological findings were markedly different.

Rickets is a type of metabolic bone disease affecting young, growing animals and is often characterized by decreased growth rate, lameness, and angular limb deformities [16]. It is caused by inadequate nutrition, renal disease, or genetic defect(s), resulting in reduced mineralization of cartilage during endochondral ossification of newly established organic bone matrix [16]. In domestic animals, rickets is primarily caused by vitamin D or phosphorus deficiencies [16]. Additionally, genetic causes of rickets have been reported in dogs, cats, pigs, and sheep, including vitamin D-dependent rickets type I (VDDR I), vitamin D-resistant rickets type II (VDRR-II), and hypophosphatemic rickets [16]. Rickets has been reported in wildlife species; however, most cases involve animals raised under human care, as has been reported in polar bear cubs (*Ursus maritimus*) [17], chimpanzees (*Pan troglodytes*) [18], a white-faced saki (*Pithecia pithecia*) [19], and a crab-eating fox (*Cerdocyon thous*) [20], all of which involved husbandry deficiencies. There have been some exceptions, including reported cases of speculated rickets in wild arctic foxes (*Alopex lagopus*) [21] and a wild crab-eating hawk (*Buteogallus aequinoctialis*) [22].

Chondrodysplasia includes inherited diseases caused by abnormal development of cartilage and is often characterized by disproportionate dwarfism [23]. Genetic defects in the formation of collagen, proteoglycans, and signal transduction mechanisms such as fibroblast growth factor 3 (FGFR3) deficiency have been identified in several types of chondrodysplasias in humans [23]. Chondrodysplasia of Alaskan Malamutes is characterized as a delayed and irregular endochondral bone formation with clinical, radiographic, and histopathologic changes similar to what is seen in rickets [24]. Although chondrodysplasia and disproportionate dwarfism are well documented in humans and domestic animals, including multiple breeds of dogs, sheep, goats, and cattle [25], it is rare in wildlife [26,27,28]. Reported cases in free-ranging animals include a red deer (*Cervus elaphus*) [29], an Asian elephant (*Elephas maximus*) [26], a Nubian giraffe (*Giraffa camelopardalis camelopardalis)* [27], an Angolan giraffe (*Giraffa giraffa angolensis*) [27], and a Seba’s short-tailed bat (*Carollia perspicillata*) [28].

Osteoporosis is the most common type of metabolic bone disease seen in humans and domestic animals [3]. It is defined as decreased bone quantity characterized by an imbalance between bone formation and resorption, resulting in bone that is structurally normal but has reduced strength [3]. Causes include starvation; deficiency in calcium, phosphorus, or copper; disuse; lactation; chronic cadmium exposure; severe gastrointestinal parasitism; and corticosteroid therapy [3]. Osteoporosis may go undetected in animals because the bone shape is unaltered, and lameness is usually only observed in cases of pathologic fractures [3]. Osteoporosis has been reported in dogs, pigs, sheep, cattle, and horses [3], as well as in an Asian small-clawed otter (*Aonyx cinereus*) [30] and free-ranging moose calves (*Alces alces*) [31], snowshoe hares (*Lepus americanus*) [32], and a mountain sheep (*Ovis canadensis*) [33]. To our knowledge, only one case of osteoporosis has been reported in an American black bear (*Ursus americanus*), a poorly conditioned female cub, which was speculated to be caused by malnutrition and resulted in pathologic fractures and physeal growth arrest [1].

We hypothesize that Case 1’s musculoskeletal abnormalities were likely the result of a genetic chondrodysplasia based on clinically normal siblings and our findings pre- and postmortem. His siblings were fed the same diet, showed no signs of musculoskeletal disease, and were successfully released. Nutritional rickets is considered unlikely in a well-conditioned wild animal. Furthermore, nutritional rickets should correct in 1–2 months with a balanced diet, but Case 1 declined in mobility as he grew wider and stockier instead of taller.

Case 1 had normal total and ionized calcium and phosphorus concentrations, which is not typical for nutritional or known genetic rickets, including heritable renal phosphate wasting diseases, vitamin D-dependent rickets type I, hereditary vitamin D-resistant rickets, Cyp24al (24-hydroxylase) knockout, or calcium-sensing receptor knockout mice models [16]. Furthermore, the physeal lesions seen with rickets are a consequence of hypophosphatemia, making it unlikely for an animal with adequate serum phosphorus concentrations to develop the disease [34,35]. It is possible that Case 1 had a disease similar to Alaskan Malamute chondrodysplasia, which can appear almost histologically identical to rickets and has been mistaken for vitamin D-resistant rickets [24]. Similar to Case 1, Alaskan Malamutes with dwarfism develop osseous changes despite receiving a balanced diet [24]. Additionally, these dogs tend to have serum calcium and phosphorus concentrations and ALP activity within the reference intervals established for healthy canines [24,36].

The vitamin D concentration of Case 1 was low compared to healthy black bears and dogs [15]. It is unknown what impact low vitamin D concentration had in the development of potential chondrodysplasia in Case 1. In Alaskan Malamute chondrodysplasia, mineralization of bone and vitamin D metabolism are not primary factors in pathogenesis based on normal calcium and phosphorus concentrations and normal appositional bone formation [24]. Whole-genome sequencing would be needed to confirm a genetic chondrodysplasia in Case 1 and further assess the role of vitamin D in the proposed disease process.

Furthermore, parasitism and subsequent gastrointestinal disease may play a role in malabsorption of nutrients. It is unknown what impact the enterocolitis and *Capillaria* may have had on Case 1; however, the patient had normal protein levels, was gaining weight, and did not have gastrointestinal clinical signs. In addition, it is common for healthy free-ranging bears in this population to have some level of endoparasitism [37]. Therefore, it is less likely that it played a significant role in this animal’s musculoskeletal disease.

CT findings in Case 1 were consistent with epiphyseal dysplasia, based on the delayed ossification of all epiphyses. In dogs and cats, the most common causes of epiphyseal dysplasia are congenital hypothyroidism and mucopolysaccharidosis, characterized by delayed mineralization of the epiphyseal cartilage and cuboidal bones and by shortened vertebrae due to decreased end plate ossification [38]. Hereditary, multiple epiphyseal dysplasia of Beagles has also been reported, with similar findings [38]. Multiple epiphyseal dysplasia is a type of chondrodysplasia; thus, we suspect that Case 1 had a unique chondrodysplasia manifesting as epiphyseal dysplasia on imaging. Case 1 represents the first clinical report of disproportionate dwarfism and suspected chondrodysplasia in a juvenile American black bear (*Ursus americanus*).

We hypothesize that Case 2’s musculoskeletal abnormalities were caused by chronic malnutrition based on being found alone and emaciated, as well as the biochemistry, imaging, and necropsy findings. Impaired growth and maturation from chronic nutritional (protein or energy) deficiency may result in osteoporosis (lack of protein matrix in bone), widened joint spaces, and proportionate dwarfism; however, joint laxity is not a typical finding of nutritional osteoporosis. Malnutrition (or over-nutrition) has been speculated to result in carpal joint laxity in large-breed puppies by contributing to muscle weakness [5]. However, unlike Case 2, most of the puppies in the aforementioned study had self-limiting disease and no signs of pain [5]. Although joint laxity secondary to chronic malnutrition is not well described in veterinary medicine, joint hypermobility in humans has been found to be associated with moderate to severe malnutrition in children, and those with hypermobile joints were more likely to have musculoskeletal symptoms including pain [6]. Additionally, rat models have shown that malnutrition can result in decreased collagen synthesis [39,40]. We hypothesize that chronic malnutrition may have resulted in muscle weakness and decreased collagen formation contributing to joint laxity.

Blood work findings (panhypoproteinemia; decreased BUN, TP, and glucose concentrations; normal calcium, phosphorus, vitamin D, and thyroid hormone concentrations) suggest malnutrition, and do not support secondary hyperparathyroidism or congenital hypothyroidism, which has been reported in bears [14].

The imaging findings in Case 2 were consistent with prior trauma: there was evidence of a healing rib fracture, but no evidence of osteoporosis to suggest underlying metabolic bone disease. The intervertebral disc space collapse and epidural fat attenuation at L3–4 were concerning for disc herniation, which in such a young animal was also likely traumatic and may have contributed to the generalized pain seen in this case. Although skeletal maturity appeared slightly delayed in comparison to what is expected in a black bear of similar age, this was most likely the result of chronic malnutrition (corroborated by thin body condition, small size for age, and metaphyseal growth arrest observed histologically). The mild changes and normal appearance of the vertebrae and lack of epiphyseal stippling make epiphyseal dysplasia an unlikely differential.

Histopathological findings were consistent with chronic malnutrition and osteoporosis. Although osteoporosis was not noted on CT, histopathology is a more sensitive diagnostic modality for the detection of mild to moderate osteoporosis. Serous atrophy of medullary adipose tissue is a common feature of starvation-induced osteoporosis in animals [3]. Case 2 was emaciated on presentation, indicating sufficient time for bone marrow fat to be mobilized and replaced with hyaluronic acid-rich fluid [3]. Severe malnutrition can inhibit longitudinal bone growth, resulting in the capped metaphyseal physes seen in this case [3].

The observed degree of lameness, immobility, and joint laxity in Case 2 was unique, and an inherited collagen disorder cannot be ruled out. Collagen disorders such as Ehlers–Danlos syndrome and osteogenesis imperfecta were considered but do not fit the whole clinical picture. Furthermore, collagen in the ligaments, dermis, and vessels was histologically normal. It is possible that the subjectively thickened articular cartilage (pathogenesis undetermined) may help explain joint laxity, but the significance of this change is uncertain without comparisons to an age-matched control. Case 2 represents the first clinical report of concurrent nutritional osteoporosis, severe joint laxity, and generalized pain in an American black bear (*Ursus americanus*).

Regardless of fully confirming nutritional etiologies with these cases, an appropriate diet is essential in preventing nutrition-associated diseases in bears. Feeding appropriate formula to neonates and providing juveniles with a balanced and diverse diet that mimics the natural diet of free-ranging bears is fundamental for rehabilitation and care. Moreover, habituation to consuming human, especially discarded, foods is a significant threat to wild black bear health. Not only are bears then more likely to be euthanized due to human–bear conflicts, but they are also more likely to be over- or under-conditioned, have dental disease, or have chronic nutritional deficiencies [41,42]. Malnutrition of young bears may also occur during times of mast failure in which there is a seasonal decrease in natural food sources, causing them to starve or search out human-related foods. Wildlife agencies, parks, and towns with high concentrations of black bears should have mitigation methods in place, including public education programs, law enforcement, and animal management options (repellents, aversive conditioning, translocation, etc.) to help prevent these issues [43].

## 6. Conclusions

In conclusion, we describe diverse pathological conditions underlying a similar presentation of small stature, joint laxity, and generalized pain in juvenile free-ranging black bears. As pathogenesis remains uncertain, rehabilitators, veterinarians, pathologists, and biologists should monitor for developmental, genetic, or other causes of musculoskeletal diseases. While a small juvenile bear may appear to be a “runt”, there may be more serious underlying pathologies present, especially if disproportionately statured or accompanied by generalized pain. Thorough palpation of limbs, joint range of motion, and observation of gait are important to detect these abnormalities and should be performed during routine examinations of free-ranging black bears for rehabilitation.

We continue to collect data on vitamin D profiles in black bears for future research and continue to screen for abnormal skeletal characteristics radiographically. Further research is needed to determine normal musculoskeletal histopathology in juvenile black bears. Practitioners who come across similar cases should consider examining tendons and ligaments for collagen content and crosslinking, undertaking electron microscopy to examine collagen structure, and performing whole-genome sequencing to determine a genetic cause for the disease.

## Figures and Tables

**Table 1:** Clinically relevant complete blood count and plasma biochemistry results from Case 1, Case 2, and healthy 11–12-month-old American black bears (*Ursus americanus*; *n* = 17) upon release from rehabilitation.

Parameter (unit)	Case 1 Intake	Case 1 Recheck	Case 2 Intake	Case 2 Recheck	Healthy Bears Median (Min–Max) [13]
White blood cell count (×10^3^/µL)	10.8	-	3.0	-	13.90 (7.50–28.50)
Red blood cell count (×10^6^/µL)	6.7	-	5.99	-	9.26 (7.38–10.61)
Hematocrit (%)	32.5	-	26.4	-	48.20 (37.20–53.60)
Absolute neutrophils (×10^3^/µL)	7.99	-	1.71	-	11 (5.72–25.11)
Absolute lymphocytes (×10^3^/µL)	1.81	-	1.05	-	1.79 (1.12–2.93)
Absolute monocytes (×10^3^/µL)	0.39	-	0.19	-	0.46 (0.22–1.08)
Absolute eosinophils (×10^3^/µL)	0.58	-	0.04	-	0.56 (0.21–1.69)
Absolute basophils (×10^3^/µL)	0.01	-	0.01	-	-
Platelets (×10^3^/µL)	367	-	678	-	479 (22–622)
Blood urea nitrogen (mg/dL)	8	15	8	11	21 (11–38)
Creatinine (mg/dL)	0.5	0.4	0.8	0.5	1.10 (0.80–1.30)
Total protein (g/dL)	4.7	7.6	4.0	5.0	6.90 (6–8)
Albumin (g/dL)	2.3	3.2	2.3	2.9	3.80 (3.50–4.30)
Globulin (g/dL)	2.4	4.4	1.7	2.1	2.90 (2.40–4.20)
Glucose (mg/dL)	148	163	116	81	164 (132–200)
Total calcium (mg/dL)	8.5	10.1	7.1	9.5	8.90 (6.20–9.90)
Phosphorus (mg/dL)	4.2	3.8	3.5	5.0	6.60 (3.00–7.90)
Alkaline phosphatase (µ/L)	26	215	21	41	84 (42–291)
Alanine aminotransferase (µ/L)	15	17	12	42	35 (22–100)
Aspartate aminotransferase (µ/L)	53	38	62	121	87 (52–271)
Creatine kinase (µ/L)	441	316	53	89	241 (172–998)

**Table 2:** Vitamin D profile results from Case 1 upon intake, Case 2 upon euthanasia, and American black bears (*Ursus americanus*) 11–18 months old (median age 11 months, *n* = 14) upon release without clinical signs of metabolic bone disease or malnutrition.

Parameter (Unit)	Case 1 Intake	Case 2 Euthanasia	Systemically Healthy Bears Median (Range)
25-Hydroxyvitamin D (nmol/L)	9	25	25 (13–46)
Ionized calcium (mmol/L)	1.28	1.32	1.2 (1.1–1.3)
Parathyroid hormone (pmol/L)	8.6	1.7	11.8 (3–24.7)

## Data Availability

Data are unavailable due to medical records being protected under HIPAA regulations.

## Supplementary Materials

The following supporting information can be downloaded at: https://www.mdpi.com/article/10.3390/ani14142088/s1, Video S1. Case 1: Hyperpronation and hypersupination of forelimbs noted during recheck examination with manipulation approximately 180 degrees in each direction. Video S2. Case 1’s clinically normal sibling: Note lack of forelimb joint laxity when manipulated similarly to Case 1.

## References

[1] Sheldon J.D., Cordero-Aponte C., Reibel V., Blair C.D., Zhu X., Gerhold R., Cushing A., Ramsay E.C., Dodd D., Dennis M. (2022). Morbidity and mortality of FREE-ranging american black bears (*Ursus americanus*) undergoing rehabilitation in Eastern Tennessee, USA, 1996–2021. J. Wildl. Dis..

[2] Dittmer K.E., Morley R.E., Smith R.L. (2017). Skeletal deformities associated with nutritional congenital rickets in newborn lambs. N. Z. Vet. J..

[3] Craig L.E., Dittmer K.E., Thompson K.G., Maxie M.G. (2016). Chapter 2—Bones and Joints. Jubb, Kennedy & Palmer’s Pathology of Domestic Animals.

[4] Dubail J., Cormier-Daire V. (2021). Chondrodysplasias with Multiple Dislocations Caused by Defects in Glycosaminoglycan Synthesis. Front. Genet..

[5] Cetinkaya M.A., Yardimci C., Sağlam M. (2007). Carpal laxity syndrome in forty-three puppies. Vet. Comp. Orthop. Traumatol..

[6] Hasija R.P., Khubchandani R.P., Shenoi S. (2008). Joint hypermobility in Indian children. Clin. Exp. Rheumatol..

[7] Bull E.L., Torgersen T.R., Wertz T.L. (2001). The importance of vegetation, insects, and neonate ungulates in black bear diet in northeastern Oregon. Northwest Sci..

[8] Benson J.F., Chamberlain M.J. (2006). Food Habits of Louisiana Black Bears (*Ursus americanus luteolus*) in Two Subpopulations of the Tensas River Basin. Am. Midl. Nat..

[9] Powell R.A., Zimmerman J.W., Seaman D.E. (1997). Ecology and Behaviour of North American Black Bears: Home Ranges, Habitat, and Social Organization.

[10] Hamilton R.J., Marchinton R.L. (1980). Denning and Related Activities of Black Bears in the Coastal Plain of North Carolina. Bears Their Biol. Manag..

[11] Garshelis D.L., Scheick B.K., Doan-Crider D.L., Beecham J.J., Obbard M.E. (2016). Ursus americanus, American Black Bear.

[12] Lee D.J., Vaughan M.R. (2004). Black Bear Family Breakup in Western Virginia. Northeast Nat..

[13] Mayhew A., Giori L., Zhu X., Sheldon J.D. (2024). Hematology and plasma chemistry comparisons among juvenile american black bears (*Ursus americanus*) undergoing rehabilitation. J. Zoo Wildl. Med..

[14] Duncan R.B., Jones J.C., David Moll H., Moon M.M., Blodgett D.J., Vaughan M.R. (2002). Cretinism in a North American Black Bear (*URSUS americanus*). Vet. Radiol. Ultrasound.

[15] Michigan State University Veterinary Diagnostic Laboratory Endocrinology Reference Intervals. https://cvm.msu.edu/assets/documents/VDL/Endocrinology-Reference-Ranges.pdf.

[16] Dittmer K.E., Thompson K.G. (2011). Vitamin D metabolism and rickets in domestic animals: A review. Vet. Pathol..

[17] Kenny D.E., Irlbeck N.A., Eller J.L. (1999). Rickets in Two Hand-Reared Polar Bear (*Ursus maritimus*) Cubs. J. Zoo Wildl. Med..

[18] Junge R.E., Gannon F.H., Porton I., McAlister W.H., Whyte M.P. (2000). Management and prevention of Vitamin D deficiency rickets in captive-born juvenile chimpanzees (*Pan troglodytes*). J. Zoo Wildl. Med..

[19] Minich D.J., Henry B.A., Levens G.P. (2022). Metabolic bone disease in a white-faced saki (*Pithecia pithecia*). Vet. Rec. Case Rep..

[20] Anselmo A.M., Saldanha A., Muelbahuer E., Buch D., Reffatti De Oliveira M., Passerino A.S.M., Lange R.R., Froes T.R. (2020). Rickets in a Crab-eating Fox (*Cerdocyon thous*). Acta Sci. Vet..

[21] Conlogue G.J., Foreyt W.J., Hanson A.L., Ogden J.A. (1979). Juvenile rickets and hyperparathyroidism in the arctic fox. J. Wildl. Dis..

[22] Guerra R.R., Dias G.F., Hallamys de Lima Nascimento H., Soares de Oliveira Neto T., Lucena R. (2018). Metabolic bone diseases in a wild crab-eating hawk and a caboclo hawk in Paraiba. Acta Vet. Bras..

[23] Thompson K.G., Blair H.T., Linney L.E., West D.M., Byrne T. (2005). Inherited chondrodysplasia in Texel sheep. N. Z. Vet. J..

[24] Sande R.D., Alexander J.E., Spencer G.R., Padgett G.A., Davis W.C. (1982). Dwarfism in Alaskan malamutes: A disease resembling metaphyseal dysplasia in human beings. Am. J. Pathol..

[25] Florczuk-Kołomyja P., Gruszczynska J. (2016). Genetic background of chondrodysplasia in domestic dog (*Canis lupus familiaris*)—In silico analysis. Acta Sci. Pol. Zootech..

[26] de Silva S., Weerathunga U.S., Pushpakumara T.V. (2014). Morphometrics and behavior of a wild Asian elephant exhibiting disproportionate dwarfism. BMC Res. Notes.

[27] Brown M.B., Wells E. (2020). Skeletal dysplasia-like syndromes in wild giraffe. BMC Res. Notes.

[28] Carneiro L., Mellado B., Monteiro L.R., Nogueira M.R. (2023). Dwarfism in a Seba’s short-tailed bat, *Carollia perspicillata*, with comments on its flight aerodynamics. Can. J. Zool..

[29] Simpson J.W., Else R.W., Butowski D., Fletcher T.J. (2011). Dwarfism associated with chondrodysplasia in a red deer (*Cervus elaphus*). Vet. Rec..

[30] Kim I.-S., Sim J.-H., Cho J.-W., Kim B., Lee Y., Ahn D. (2020). Osteoporosis in an Asian small-clawed otter (*Aonyx cinereus* Illiger, 1815). J. Vet. Med. Sci..

[31] Ytrehus B., Skagemo H., Stuve G., Sivertsen T., Handeland K., Vikøren T. (1999). Osteoporosis, bone mineralization, and status of selected trace elements in two populations of moose calves in norway. J. Wildl. Dis..

[32] Amuno S., Al Kaissi A., Jamwal A., Niyogi S., Quenneville C.E. (2018). Chronic arsenicosis and cadmium exposure in wild snowshoe hares (*Lepus americanus*) breeding near Yellowknife, Northwest Territories (Canada), part 2: Manifestation of bone abnormalities and osteoporosis. Sci. Total Environ..

[33] Bleich V.C., Stahmann J.G., Bowyer R.T., Blake J.E. (1990). Osteoporosis and Cranial Asymmetry in a Mountain Sheep (*Ovis canadensis*). J. Wildl. Dis..

[34] Sabbagh Y., Carpenter T.O., Demay M.B. (2005). Hypophosphatemia leads to rickets by impairing caspase-mediated apoptosis of hypertrophic chondrocytes. Proc. Natl. Acad. Sci. USA.

[35] Miedlich S.U., Zalutskaya A., Zhu E.D., Demay M.B. (2010). Phosphate-induced apoptosis of hypertrophic chondrocytes is associated with a decrease in mitochondrial membrane potential and is dependent upon Erk1/2 phosphorylation. J. Biol. Chem..

[36] Fletch S., Smart M., Pennock P., Subden R. (1973). Clinical and Pathologic Features of Chondrodysplasia in the Alaskan Malamute. J. Am. Vet. Med. Assoc..

[37] Riese K.E. (2023). Black Bear Population Health Monitoring in the Southeast. Master’s Thesis.

[38] Pollard R.E., Phillips K.L., Thrall D.E. (2018). Chapter 18—Orthopedic Diseases of Young and Growing Dogs and Cats. Textbook of Veterinary Diagnostic Radiology.

[39] Spanheimer R., Zlatev T., Umpierrez G., DiGirolamo M. (1991). Collagen production in fasted and food-restricted rats: Response to duration and severity of food deprivation. J. Nutr..

[40] Rao J.S., Rao V.H. (1980). Effect of protein malnutrition on the metabolism of dermal collagen in the rat. Ital. J. Biochem..

[41] Beckmann J.P., Berger J. (2003). Rapid ecological and behavioural changes in carnivores: The responses of black bears (*Ursus americanus*) to altered food. J. Zool..

[42] Hatch K.A., Kester K.A., Loveless A., Roeder B.L., van Manen F.T. (2022). Tooth wear and the apparent consumption of human foods among American black bears (*Ursus americanus*) in Great Smoky Mountains National Park, USA. Mamm. Biol..

[43] Lackey C.W., Breck S.W., Wakeling B.F., White B. (2018). Human–Black Bear Conflicts.

